# Quantitative Detection of Tank Floor Defects by Pseudo-Color Imaging of Three-Dimensional Magnetic Flux Leakage Signals

**DOI:** 10.3390/s23052691

**Published:** 2023-03-01

**Authors:** Zhijun Yang, Jiang Yang, Huaiqing Cao, Han Sun, Yazhong Zhao, Bowen Zhang, Changpeng Meng

**Affiliations:** School of Mechanical Science and Engineering, Northeast Petroleum University, Daqing 163318, China

**Keywords:** three-dimensional (3D) magnetic sensor, magnetic flux leakage, pseudo-color imaging, quantitative identification, least-squares support-vector machine

## Abstract

Highly integrated three-dimensional magnetic sensors have just been developed and have been used in some fields, such as angle measurement of moving objects. The sensor used in this paper is a three-dimensional magnetic sensor with three Hall probes highly integrated inside; 15 sensors are used to design the sensor array and then measure the magnetic field leakage of the steel plate; the three-dimensional component characteristics of the magnetic field leakage are used to determine the defect area. Pseudo-color imaging is the most widely used in the imaging field. In this paper, color imaging is used to process magnetic field data. Compared with analyzing the three-dimensional magnetic field information obtained directly, this paper converts the magnetic field information into color image information through pseudo-color imaging and then obtains the color moment characteristic values of the color image in the defect area. Moreover, the least-square support-vector machine and particle swarm optimization (PSO-LSSVM) algorithm are used to quantitatively identify the defects. The results show that the three-dimensional component of the magnetic field leakage can effectively determine the area range of defects, and it is feasible to use the color image characteristic value of the three-dimensional magnetic field leakage signal to identify defects quantitatively. Compared with a single component, the three-dimensional component can effectively improve the identification rate of defects.

## 1. Introduction

A storage tank is mainly made of welded ferromagnetic steel plates. Due to the long-term storage of corrosive chemical media such as petroleum, its bottom plate will be corroded to different degrees, resulting in defects of different degrees of damage. Non-destructive testing of the tank bottom plate is crucial for the integrity evaluation of the storage tank [1]. At present, the non-destructive testing methods for ferromagnetic steel plates mainly include magnetic powder [2], penetration [3], guided wave [4,5], acoustic emission [6], magnetic leakage [7,8,9], ultrasound [10], etc. Magnetic leakage testing technology, for example, has been widely used in the detection of ferromagnetic materials like storage tank bottom plates and pipelines [11]. After magnetizing the ferromagnetic material, the material at the defect depends strongly on magnetic field strength—every gradient in permeability produces magnetic field leakage (MFL). Because the permeability of air is much lower than that of ferromagnetic material, the magnetic field will spill into the air, so detection can be accomplished by measuring the spilled magnetic field leakage [12,13,14,15,16].

MFL signal can be decomposed into three components in space, and all three components are conducive to detection. The literature [15] shows that the characteristics of the three-dimensional component are as follows: the axial component has the peak value, the radial component and the circumferential component have the peak-peak value and the zero-crossing value, and the combination of the three-dimensional magnetic leakage component can improve the detection rate.

The literature [17] has analyzed the three-dimensional magnetic flux leakage signal of the ferromagnetic tank bottom plate through a finite element simulation experiment, determined the defect contour edge according to the characteristics of the three-dimensional magnetic flux leakage component, and then established the relationship between the three-dimensional component average strength and the length, width, and depth of the defect, to realize the defect imaging. Compared with the defect imaging effect of the one-dimensional magnetic leakage component, it shows that the three-dimensional magnetic leakage component is very effective in the estimation of the defect profile and depth. Through finite element simulation experiments, a study [18] realizes the reconstruction of a 3D defect profile by using a 3D magnetic leakage signal of the ferromagnetic pipeline. A pipeline inspection gauge is used to measure the axial component reconstruction defect profile. After comparing the results of the two prediction profiles, it is concluded that the three-dimensional component’s prediction results are more accurate, indicating that the combination of the three-dimensional component is more conducive to defect analysis.

The experimental measurements of ferromagnetic materials are presented in this paper. Instead of the traditional three one-dimensional magnetic sensors, a three-dimensional magnetic sensor with a high integration degree is used to measure the three-dimensional magnetic leakage component. The sensor is a three-dimensional magnetic sensor with three Hall probes highly integrated inside. The actual measured original magnetic leakage signal contains a lot of noise signals, which will reduce the identification of the useful signals that we need. Many studies have been done on the noise reduction of MFL signals.

Songling Huang [19] implements wavelet denoising in DSP signal processing systems, which can significantly improve the signal-to-noise ratio. That is, wavelet denoising can effectively reduce noise. The literature [20] has used wavelet transform decomposition and reconstruction technology to denoise the original data and achieve an effective denoising effect. In the literature [16], wavelet denoising technology is adopted to achieve an obvious denoising effect when dealing with the defective MFL signal of steel wire rope. In this paper, the wavelet transform technique is used to denoise the original MFL signal collected in the experiment, decompose the original signal, combine it with soft threshold filtering, and finally reconstruct the signal.

The magnetic leakage signals corresponding to different surface damage of ferromagnetic materials are very different. The damage degree of defects can be classified according to the characteristics of the magnetic leakage signals. In the aspect of the classification algorithm, the research on the support vector machine and neural network is more in-depth and more widely used. The literature [21] has studied the relationship between the axial crack size of ferromagnetic pipes and the strength of the axial leakage magnetic field component of defects. It used this relationship to carry out the classification of defects. In the literature [22], support vector machines have been used to classify the thickness of ferromagnetic steel plates, and experiments proved that support vector machines could obtain effective classification results. In the literature [23], neural networks have been used to classify the defects of ferromagnetic pipes and good classification results were obtained.

Fifteen highly integrated three-dimensional magnetic sensors are designed into a sensor array, and the magnetic field data measured by the sensor array are processed by color imaging. This paper uses the least-squares support-vector machine algorithm to classify the defects. The corresponding color image was obtained using the 3D magnetic leakage data after noise reduction, and then the color moment value of the defect area was obtained. Finally, the color moment value was fed into the least-squares support-vector machine to compare the defect classification effects of the three-dimensional magnetic field leakage and the single-dimensional magnetic field leakage.

The highly integrated three-dimensional magnetic sensor used in this paper has some suggestions for the development of magnetic leakage testing instruments. The three-dimensional magnetic field component information is converted into color image information by pseudo-color imaging, and then the color moment of defects is extracted to perform the quantitative recognition of defects, which has a certain reference for the quantitative recognition of the damage degree of the steel plate. 

The remainder of this paper is organized as follows: the Section 2 introduces the experimental equipment used in this paper, the Section 3 introduces the original data processing method, and the Section 4 introduces the quantitative identification method of defects. The Section 5 contains the results and analysis of the experiments.

## 2. Experimental Setup

### 2.1. The Overall Structure of the Experimental Device

**Figure 1:**
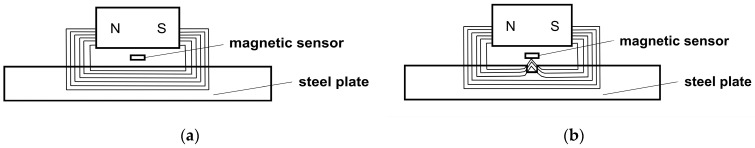
Schematic diagram of the magnetic flux leakage detection principle. (**a**) no defect; (**b**) defective.

The three-dimensional magnetic flux leakage signal detection system for the tank bottom plate designed in this experiment is shown in Figure 1, including the permanent magnet magnetization structure, the three-dimensional magnetic sensor array, the walking mechanism, the data acquisition module, and a data processing module.

### 2.2. Principle of Magnetic Flux Leakage Detection

The tank bottom plate is made of Q35 steel. When there are no defects on its upper or lower surface, the magnetic field intensity in the air on the upper surface of the tank bottom plate is low and relatively uniform. If there is a defect on the upper surface of the tank floor, then the permeability at the defect’s vicinity will decrease due to magnetic leakage caused by air gaps or cracks, and the magnetic circuit will be changed accordingly. The magnetic field will overflow the air at the defect and form a magnetic field leakage. The magnetic field leakage in the air above the defect is strong and fluctuates greatly. The magnetic sensor captures the magnetic field leakage, and the defect of the ferromagnetic steel plate can be determined according to the characteristics of the magnetic leakage signal.

### 2.3. Magnetization Device

In this paper, the thicknesses of the ferromagnetic steel plate are 4, 6, 8, 10, 12, and 13 mm. Table 1 shows the magnetization structure size used in this paper based on the finite element software analysis. The magnetization structure comprises a pole shoe, an armature, and a permanent magnet. The permanent magnet’s length, width, and height are 160 mm, 36 mm, and 15 mm, respectively; the pole boot’s length, width, and height are 160 mm, 36 mm, and 30 mm, respectively; and the armature’s length, width, and height are 160 mm, 200 mm, and 30 mm, respectively. The distance between the magnetized structure and the ferromagnetic steel plate to be tested, i.e., the lift value of the magnetization device, is maintained at 1.5 mm.

### 2.4. Three-Dimensional Magnetic Sensor Module

**Figure 2:**
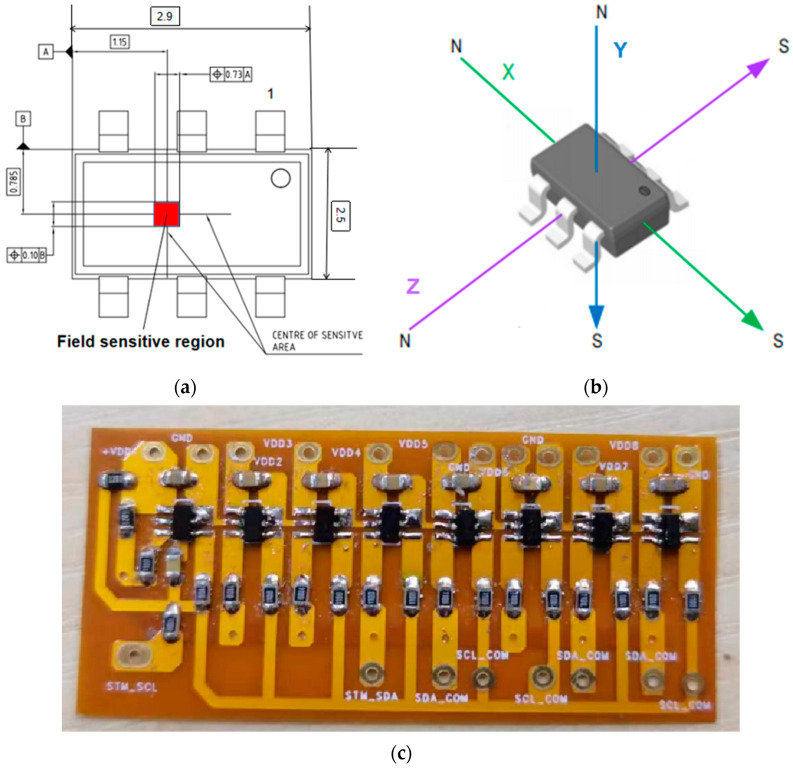
3D magnetic sensor. (**a**) Three-dimensional magnetic sensor size diagram; (**b**) 3D magnetic sensor physical picture; (**c**) Three-dimensional magnetic sensor array.

Three uniaxial magnetic sensors are used to form a three-dimensional magnetic sensing unit. For example, in the literature [14], three Hall elements are used to collect information on three-dimensional magnetic field leakages, respectively. Then, a three-dimensional magnetic field leakage in a certain space is obtained through subsequent data processing. Different from traditional methods, the highly integrated three-dimensional magnetic sensor is adopted in this paper, and its related parameters are shown in Table 2. It integrates magnetic field probes into the same sensor chip in three directions in space. The magnetic sensitive area of the three-dimensional magnetic field probe is shown in Figure 2a, and the dimensions indicated there are in mm, so the lift values of the three-axis magnetic field are the same. Eight three-dimensional magnetic sensors are arranged horizontally to form a three-dimensional magnetic sensor array. Two such three-dimensional magnetic sensor arrays are used to form the three-dimensional magnetic sensor module in this experiment. The three-dimensional sensor array is shown in Figure 2c. A two-layer flexible circuit board is used for the entire sensor array circuit board (FPC). The circuit board’s thickness is very thin, only 0.23 mm, which can significantly reduce the lift value between the sensor and the tested steel plate. The sensor array attaches easily to the sensor box. The distance between two adjacent magnetic field sensors is 5.4 mm, so the measuring range of the magnetic sensor module composed of 15 three-dimensional magnetic sensors is 75.6 mm. As shown in Figure 2b, for convenience, the magnetic field intensity measured by the sensor in three directions is set as the X-axis component, Y-axis component, and Z-axis component. As shown in Table 2, TLV493A1B6 has high measuring sensitivity and a wide measuring range of magnetic fields. Its sensitivity is 0.098 millit (mt)/bit, and its linear measuring range of magnetic field in x, y, and z directions is −130 mt to 130 mt.

The output analog signal of the Hall element used in the literature [14] cannot be directly processed by the main control unit. Additional signal conditioning is needed between the Hall element and the main control unit to convert the analog signal into a digital signal. Moreover, a three-dimensional magnetic flux leakage signal at the same position in space needs to be obtained by three Hall elements. This will increase the complexity of the measuring device and reduce the operability of the experiment. Unlike traditional Hall components, which measure one-dimensional magnetic fields, the TLV493A1B6 outputs digital signals rather than analog signals, further simplifying the subsequent circuit design and reducing the experimental setup’s complexity. The 3D magnetic flux leakage signal output by TLV493A1B6 used in this paper is a digital signal. It communicates with the main control unit through a serial communication interface, and the output rate is up to 1 Mbit/s, reducing the measurement device’s complexity and improving the experiment’s operability.

### 2.5. Control Section

**Figure 3:**
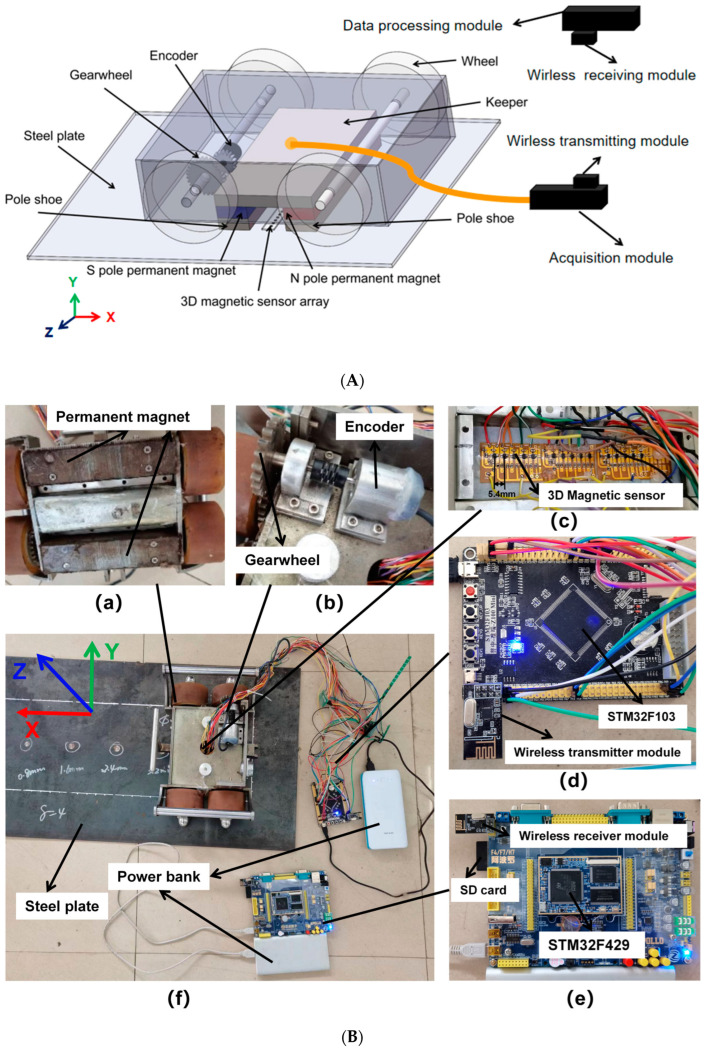
Experimental setup. (**A**) Schematic diagram of the experimental apparatus; (**B**) the actual picture of the experimental device.

As shown in Figure 3, the walking mechanism mainly comprises two groups of rollers and gears. The roller rotates synchronously through the gear and encoder in the process of moving. The gear ratio is N = 1.125, the circumference of the roller is C = 250 mm, and the number of lines of the encoder is I = 600. For each pulse output by the encoder, the traveling distance of the roller can be calculated as follows: S is C/(N * I). This way, the detection device’s traveling distance can be obtained.

The data acquisition module communicates with the 3D magnetic sensor module via the serial communication bus to obtain 3D magnetic field signals and capture the encoder’s pulse data, as shown in Figure 3A. The data acquisition and processing modules are separated by a wireless transmission module and a wireless receiving module to facilitate experimental operation. The wireless transmitting module and wireless receiving module use a single chip wireless transceiver NRF24L01 working in the 2.4~2.5 gigahertz (GHz) universal frequency band. NRF24L01 has the characteristics of strong anti-interference ability and the highest working rate of 2 megabits/second (Mbps), very suitable for industrial control occasions. The NRF24L01 communicates with the data acquisition module through the Serial Peripheral Interface. The packet transmitted each time by the wireless transmitting module contains the number of pulses output by the encoder and the three-dimensional component of the leakage magnetic field.

Contains the wireless receiving module to receive the data packet sent by the wireless transmitting module. The data processing module communicates with the wireless receiving module through serial peripheral interface communication. The data processing module processes the data in the packet. When the mileage data is updated, the data processing module writes the 3D magnetic field signal data and mileage data into the data processing module’s memory card in real time.

If there is a defect in the ferromagnetic steel plate, the location and size of the defect can be determined using the encoder’s length and the sensor array’s three-dimensional magnetic field signal.

## 3. Data Processing

**Figure 4:**
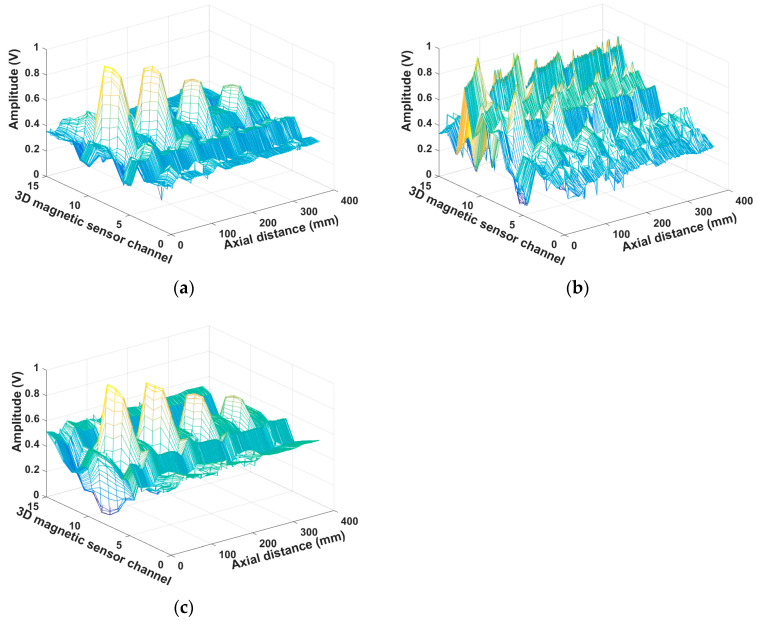
3D MFL signals of 15 channels before noise reduction. (**a**) x axis before noise reduction; (**b**) y axis before noise reduction; (**c**) z axis before noise reduction.

**Figure 5:**
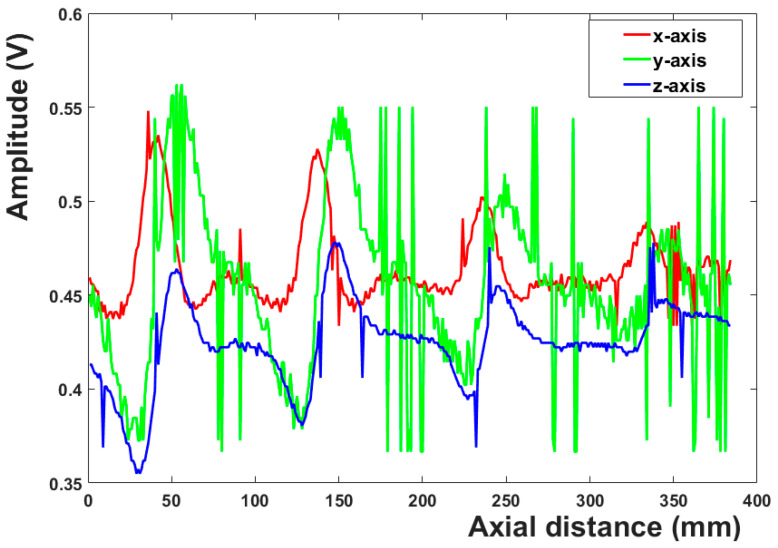
3D magnetic leakage before noise reduction in channel 8.

The magnetic leakage signals of 15 channels of ferromagnetic steel plate with a thickness of 4 mm are shown in Figure 4. Figure 4a is the magnetic leakage signal of the X-axis, Figure 4b is the magnetic leakage signal of the Y-axis, and Figure 4c is the magnetic leakage signal of the Z-axis. The magnetic leakage signal of the eighth channel is shown in Figure 5. The original data collected not only contains the magnetic flux leakage signal captured by the sensor but also contains a lot of noise signals, which mainly include the electromagnetic noise on the circuit board and the noise caused by the slight change of the lifting value during the movement of the detection device. As shown in Figure 4, noise signals blur the signal characteristics of defects, and some minor defect signals may even be submerged. As illustrated in Figure 5, noise signals cause many burrs in the magnetic leakage signal curve. The characteristics of effective signals will become fuzzy, difficult to identify, and inconvenient for subsequent data processing due to these useless noise signals. The acquired three-dimensional magnetic field data should be denoised to improve the localization accuracy and defect-recognition rate.

### 3.1. Filtering Using 1D Standard Widget Toolkit (SWT) Denoising

The SWT Denoising1D module of MATLAB [24] software is used for noise reduction. Sym function is selected as the wavelet basis function in MATLAB. Sym has good regularity and symmetry, which makes the signal reconstruction process relatively smooth and can, to some extent, reduce phase distortion in the signal analysis and reconstruction process. The Sym8 wavelet basis function was used in this study to perform five layers of wavelet decomposition on the original data. Next, soft threshold denoising was performed on the wavelet coefficient of each layer. Finally, the denoised wavelet coefficients were used for reconstruction. Discrete binary wavelet transform can be expressed as:

(1) $$\{\begin{array}{c} C_{j+1}\left(n\right)=\sum_{k\in Z}h\left(k-2n\right)C_{j}\left(k\right)\\ D_{j+1}\left(n\right)=\sum_{k\in Z}g\left(k-2n\right)C_{j}\left(k\right) \end{array}$$

The wavelet reconstruction algorithm can be expressed as follows:

(2) $$C_{j-1}\left(n\right)=\sum_{k\in Z}h\left(n-2k\right)C_{j}\left(k\right)+\sum_{k\in Z}g\left(n-2k\right)D_{j}\left(k\right)$$

where h(n) and g(n) are a pair of complementary conjugate filters determined by the wavelet function, h(n) is a low-pass filter, g(n) is a high-pass filter, and $C_{j}$ and $D_{j}$ are the approximate coefficient and detail coefficient of the signal on scale j, respectively. Soft-threshold filtering was performed on each layer of high-frequency coefficient after wavelet decomposition:

(3) $$\hat{d_{l}}(n)=\{\begin{array}{cc} \mathrm{sgn}\left(d_{l}\left(n\right)\right)\left(\left|d_{l}\left(n\right)-t_{l}\right|\right) & |d_{l}\left(n\right)|>t_{l}\\ 0 & |d_{l}\left(n\right)|<t_{l} \end{array}$$

where n∈Z, $d_{l}(n)$ is the original wavelet detail coefficient of the signal, and $\hat{d_{l}}(n)$ is the wavelet detail coefficient after signal thresholding.

After soft-threshold filtering, wavelet reconstruction was performed using Equation (2) to obtain the denoised signal.

**Figure 6:**
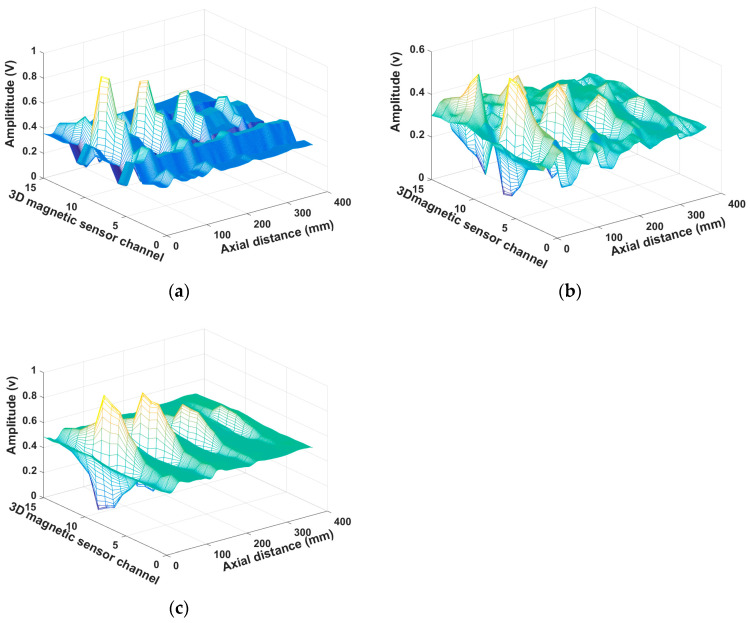
3D MFL signals of 15 channels after noise reduction. (**a**) x axis after noise reduction; (**b**) y axis after noise reduction; (**c**) z axis after noise reduction.

**Figure 7:**
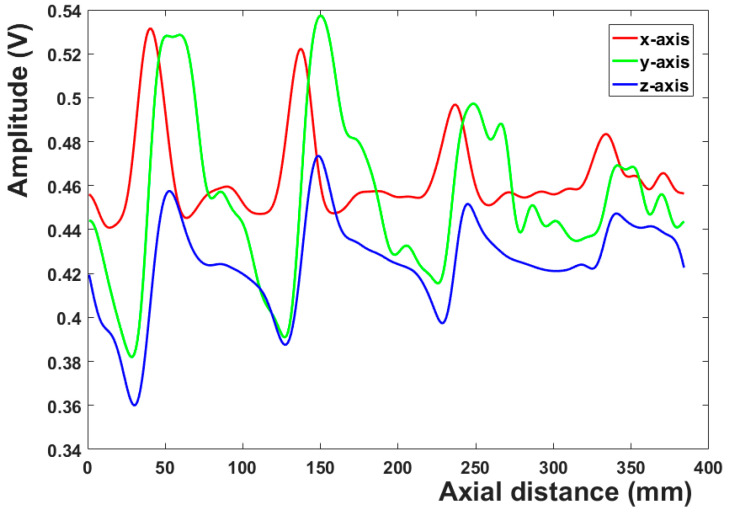
3D magnetic leakage after noise reduction in channel 8.

The three-dimensional magnetic leakage signals of 15 channels after noise reduction are shown in Figure 6a–c. The noise signals are effectively suppressed, and the characteristics of the magnetic leakage signals at the defects are very obvious. Figure 7 depicts the 3D magnetic leakage signal after noise reduction in the eighth channel. It is clear that after noise reduction, the curve of the 3D magnetic leakage signal is smooth, and the signal’s characteristics are obvious. The X-axis magnetic leakage signal will peak at the defect, and the Y-axis and Z-axis magnetic leakage signals will peak and trough. Noise reduction improves the ability to characterize defects in signals.

### 3.2. Data Interpolation

Data normalization involves the linear transformation of data to prevent “failures” to the data and improve performance. To facilitate pseudo-color imaging of the three-axis magnetic flux leakage signals, the denoised X-, Y-, and Z-axes data were normalized:

(4) $$X_{i}^{*}=\frac{X_{i}-X_{\min}}{X_{\max}-X_{\min}}$$

where $X_{i}$ denotes the initial data, $X_{\min}$ is the minimum of the initial data, $X_{\max}$ is the maximum of the initial data, and $X_{i}^{*}$ is the normalized data.

**Figure 8:**
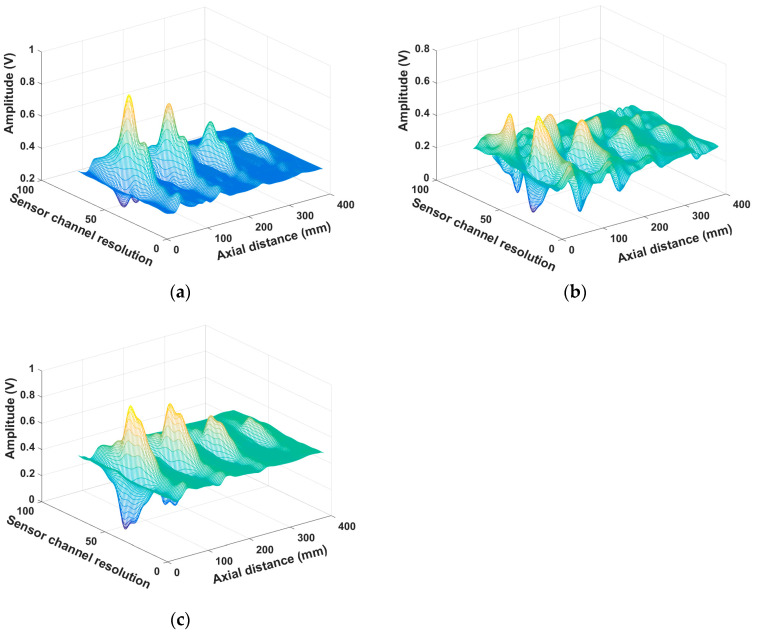
3D MFL signals after interpolation of 15 channels. (**a**) x axis after interpolation; (**b**) y axis after interpolation; (**c**) z axis after interpolation.

The data of 15 channels in the X-, Y-, and Z-axes were normalized (Figure 8). Next, cubic spline interpolation was performed on the normalized three-axis data. The signal curves after cubic spline interpolation were smoother (Figure 8a–c), indicative of stronger signal characterization ability.

### 3.3. Pseudo-Color Imaging

**Figure 9:**
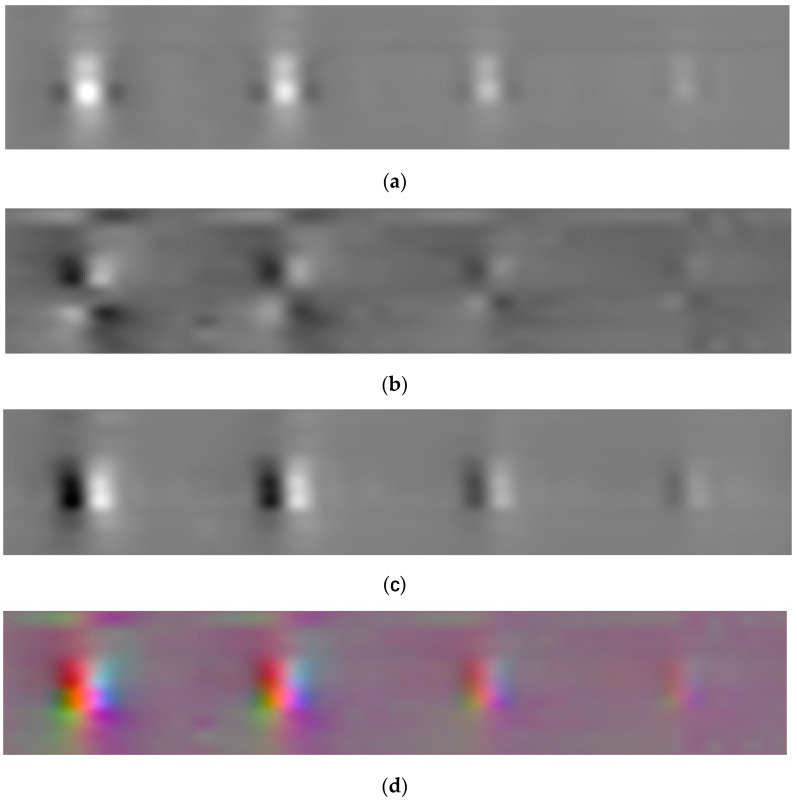
(**a**) gray image of the X-axis; (**b**) gray image of the Y-axis; (**c**) gray image of the Z-axis; (**d**) color image of the three axes.

Current magnetic flux leakage images are almost gray images; however, color images aid in defect identification. In this study, the X-, Y-, and Z-axes magnetic flux leakage signals after denoising, normalization, and cubic spline interpolation were mapped to the red, green, and blue color channels, respectively. The weights of all three color channels were 1. Therefore, a color image of the three-axis magnetic flux leakage signals was produced. When compared to gray images, the defect area in the color image was easier to distinguish from the background, and the defect signal’s characterization ability was stronger (Figure 9).

The defect area was located and segmented using pseudo-color imaging of the three-axis magnetic flux leakage signals. The eigenvalues were then calculated. After that, quantitative identification was carried out.

(1) Defect area localization and segmentation

The defect area localization and segmentation algorithm employed in this study is described as follows.

1) Based on the magnetic flux leakage signals, the red channel of the color image was chosen, and the local maximum point P among the data on the channel was obtained. P_ch_ denoted the sensor channel in which point P was located, and P_axial_ denoted the axial position of point P.
2) The two minimum points A and B closest to point P along the axial direction on sensor channel P_ch_ were determined. The center point of the defect was P, and the area range in the axial direction was |B − A|.
3) The point on the green channel corresponding to point P was denoted as P^’^, the sensor channel of P^’^ was denoted as P^’^_ch_, and the axial position of P^’^ was represented by P^’^_axial_. The minimum point A^’^ and the maximum point B^’^ closest to point P^’^ on sensor channel P^’^_ch_ along the axial direction were determined, and the axial position of B^’^ was denoted as B^’^_axial_^’^. The minimum points CH_c_ and CH_d_ closest to B^’^ at B^’^_axial_^’^ along the sensor channel direction were determined; thus, the area range of the defect in the direction of the sensor was |CH_d_ − CH_c_|.
4) The number of channels interpolated is five, and the number of color channels is three, so the color image pixel of the defect region was |B − A| × |(CH_d_ − CH_c)_ × 5| × 3.

**Figure 10:**
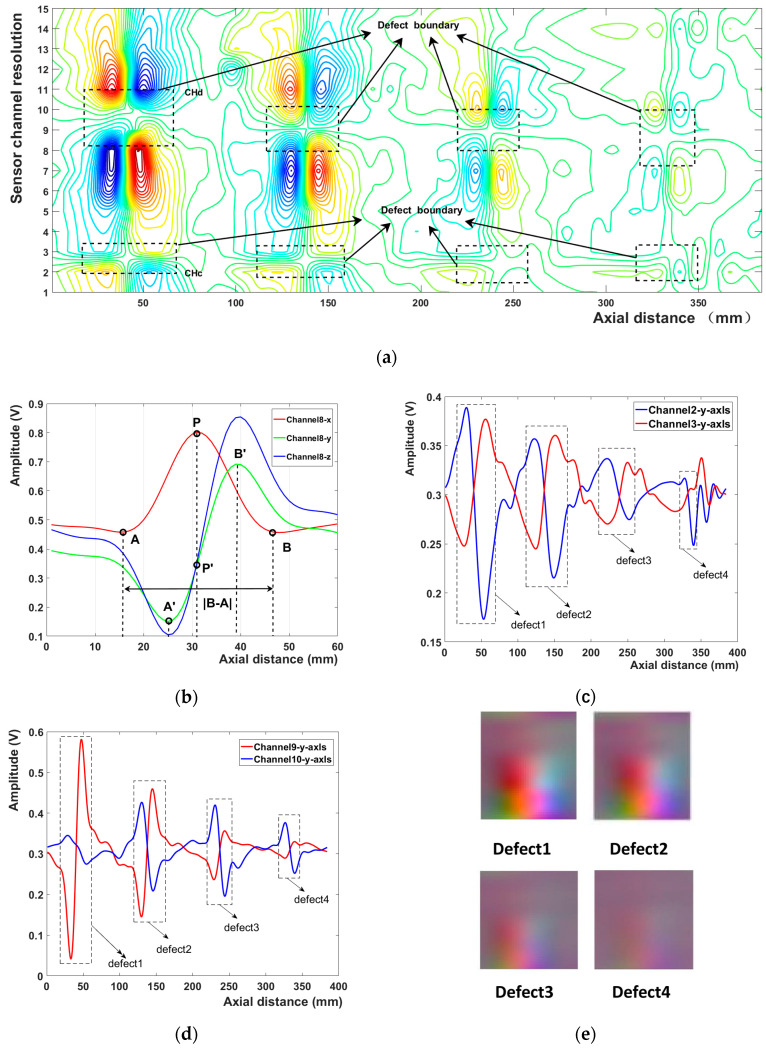
(**a**) Y-axis flux leakage signal contours for 15 sensor channels; (**b**) the first defect is in the triaxial leakage field of sensor channel 8; (**c**) Y-axis flux leakage signal curve of sensor channels 2 and 3; (**d**) Y-axis flux leakage signal curve of sensor channels 9 and 10; (**e**) four defect magnetic flux leakage color images.

The three-axis leakage magnetic field of the first defect on sensor channel 8 is shown in Figure 10b; the red, green, and blue curves represent the X-, Y-, and Z-axes leakage magnetic field signals, respectively. Figure 10a illustrates a contour map of the Y-axis magnetic flux leakage signals of the 15 sensor channels. The defect area is evident from the changes in the color of the contour lines at the boundary. Figure 10b,c depict the Y-axis magnetic flux leakage signal curves of sensor channels 2, 3, and 9, 10, respectively, showing reversed polarity at the defect. For sensor channels 2 and 10, a valley appeared after a peak at the defect, whereas for sensor channels 3 and 9, a valley appeared first, followed by a peak. Figure 10 depicts the magnetic flux leakage color images of the four defects obtained through localization and segmentation (e).

(2) Color moment extraction

The color moment method, proposed by Stricker and Orengo [25], is a simple and effective method for representing color features. The color moment effectively represents the color distribution in the image; spatial color quantization is not required, and the feature vector dimension is small. The color moment consists of the first-order (mean), second-order (variance), and third-order moments (skewness). Because color information is mainly distributed in low-order moments, the first-, second-, and third-order moments are sufficient to express the color distribution of an image. The three color moments are defined as follows:

(5) $$\{\begin{array}{c} \mu_{i}=\frac{1}{N}\sum_{j=1}^{N}P_{i,j}\\ \sigma =\sqrt{\frac{1}{N}\sum_{j=1}^{N}{\left(P_{i,j}-\mu_{i}\right)}^{2}}\\ S_{i}=\sqrt[{3}]{\frac{1}{N}\sum_{j=1}^{N}{\left(P_{i,j}-\mu_{i}\right)}^{3}} \end{array}$$

where *N* is the sum of pixels in the image, and *P_i,j_* is the *i*-th color component of the *j*-th pixel in the image.

The first three color moments of the color image of the three-axis magnetic flux leakage signals constitute a nine-dimensional color eigenvector:

(6) $$F=\left[\mu_{x},\sigma_{x},S_{x};\mu_{y},\sigma_{y},S_{y};\mu_{z},\sigma_{z},S_{z}\right]$$

The color characteristic values of four defects on the surface of a steel plate with a thickness of 4 mm in this experiment are shown in Table 3.

## 4. PSO-LSSVM to Realize Quantitative Defect Identification

The LSSVM method is used for mapping the samples to the high-dimensional feature space by using a nonlinear function, thereby transforming the nonlinear function estimation problem in the original sample space into a linear problem in the high-dimensional feature function. LSSVM is suitable for classification problems with limited samples because of its short training time and strong generalization ability. LSSVM has two important parameters: the regularization parameter and the kernel parameter. In this paper, regularization parameters and kernel parameters are taken as particles in the particle swarm optimization algorithm, and the particle swarm optimization algorithm (PSO) is used to optimize the regularization parameters and kernel parameters of LSSVM to find the optimal combination of parameters to improve the classification accuracy of LSSVM.

In this study, H is the ratio of defect depth to steel plate thickness; it is an important parameter for characterizing defects. The manually processed defects were divided into four categories: 20%, 40%, 60%, and 80%. After defect region localization and segmentation, the three color moments extracted from the X-, Y-, and Z-axes were selected as the characteristic quantity. On the upper surface of steel plates of various thicknesses, 42 defects of various shapes were manually processed. The shapes of the defects were hemispherical, cone frustum, cylindrical, or threaded hemispherical. The numbers of defects with an H value of 80%, 60%, 40%, and 20% were 11, 11, 11, and 10, respectively. These defect samples were divided into two groups: training samples and test samples. Five samples were used for each of the four types of defects. The relevant parameters of the defects are presented in Table 4 and Table 5.

The color moment eigenvalues of the single-axis magnetic flux leakage signal and the three-axis magnetic flux leakage signals at the defect were used as the input of PSO-LSSVM, and the recognition rates of the four types of defects were selected as the output. The specific steps are as follows.

(1) The minimum output coding scheme was used to encode the multiclass classification task into multiple binary classifiers.

(2) To determine the best combination of parameters, PSO was used to optimize the regularization parameter gam and the kernel parameter sig2 of LSSVM. The specific implementation steps are as follows:

1) Parameters related to initializing particles: particle swarm size, random location, velocity;
2) Evaluate the initial adaptation value of each particle;
3) Take the initial adaptation value as the current global optimal value and record the current position as the local optimal position;
4) Take the optimal adaptation value as the current global optimal value and record the current position;
5) Calculate and evaluate the fitness of particles, and update if the fitness is better;
6) Find the optimal combination of gam and sig2 parameters;
7) Repeat 4)–6) until the maximum number of iterations is reached, and output gam and sig2.

**Figure 11:**
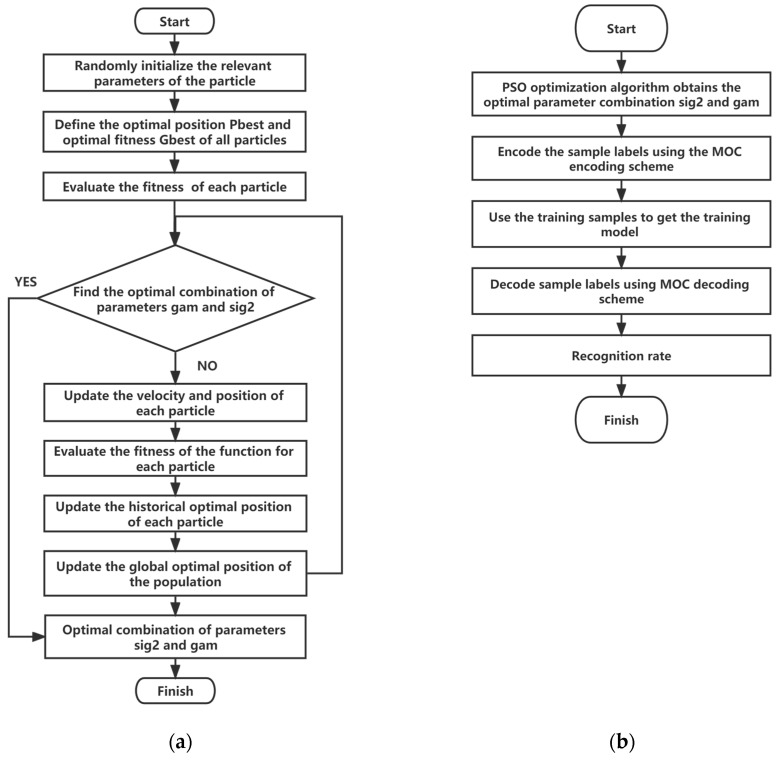
(**a**) Using particle swarm optimization (PSO) to optimize the regularization parameter gam and kernel parameter sig of LSSVM to find the optimal combination of parameters; (**b**) PSO-LSSVM flow chart.

The algorithm is depicted in Figure 11a, and the optimization results of gam and sig2 are shown in Table 6.

**Figure 12:**
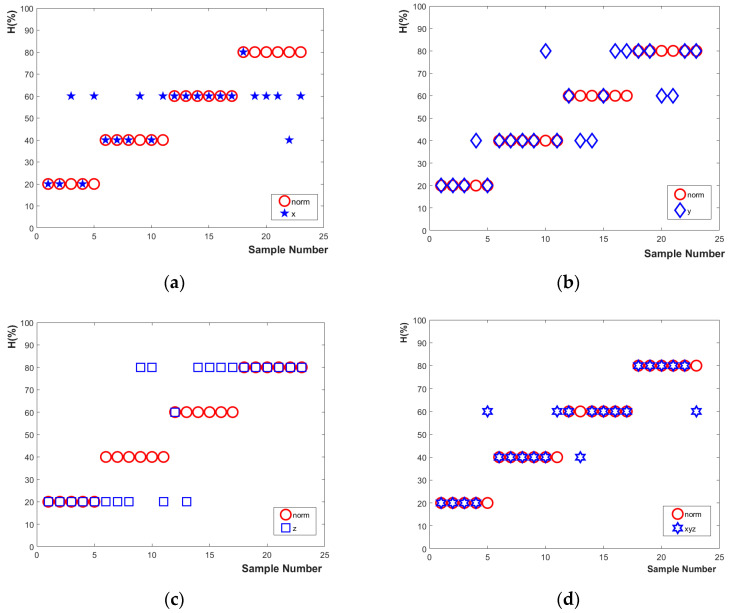
(**a**) Identification results of X-axis color moment eigenvalues; (**b**) identification results of eigenvalues of Y-axis color moments; (**c**) identification results of eigenvalues of Z-axis color moments; (**d**) identification results of X-axis, Y-axis, and Z-axis color moment eigenvalues.

(3) The radial basis function kernel RBF_kernel was adopted as the kernel function of LSSVM, and the color moment eigenvalues of the single-axis and the three-axis magnetic flux leakage signals of the training samples were used as the input to train the LSSVM model. The algorithm is depicted in Figure 11b.

(4) The recognition rates of the four types of defects were obtained by feeding the trained LSSVM model single-axis and three-axis magnetic flux leakage signals from test samples. The recognition results are shown in Figure 12 and Table 7.

Table 7 depicts the recognition results. The defect identification rate from the single-axis magnetic flux leakage signal was significantly lower than that of the three-axis magnetic flux leakage signals. The defect identification rates of the X, Y, and Z single-axis signals and of the three-axis magnetic flux leakage signals were 60.87 %, 65.22 %, 52.17 %, and 82.61 %, respectively.

## 5. Conclusions

In this study, a three-axis magnetic flux leakage detection device for storage tank floors was designed using permanent magnets. In the proposed device, permanent magnets are employed as the magnetic excitation source. The sensor array of the device includes 15 3D magnetic sensors evenly arranged in the horizontal direction and are used to measure the X-, Y-, and Z-axes magnetic field components on the surface of the tank floor. In the experiment, wavelet soft threshold denoising was performed on the collected original signal using the Sym8 wavelet basis function. Then, the defect area was located and segmented using the X- and Y-axis magnetic field characteristics to obtain the three-axis magnetic flux leakage color image of the defect. Finally, the three color moments of the defect’s three-axis magnetic flux leakage color image were extracted and used as input to PSO-LSSVM.

Experimental results revealed that the quantitative identification of defects was achieved by pseudo-color processing of the three-axis magnetic flux leakage signal components. The recognition rate of the three-axis components was significantly higher than that of a single-axis component.

The experimental results show that the highly integrated three-dimensional magnetic sensor can effectively measure the magnetic field of a steel plate, convert the magnetic field information into color image information through pseudo-color imaging, and then extract the color moment of defects, which can effectively achieve the quantitative recognition of defects. Moreover, the recognition rate of the three-dimensional components is higher than that of the uniaxial components.

In future research, we will study more types and quantities of defect samples to further enhance the recognition rate of the proposed device.

## Figures and Tables

**Table 1:** The size of the magnetized structure.

	Material	Length/mm	Width/mm	Height/mm
Pole shoe	Pure iron	160	200	30
Armature	Pure iron	160	36	30
Permanent magnet	NeFeB	160	36	15

**Table 2:** Parameters of the 3D magnetic sensor.

Sensor Type	Magnetically Sensitive Region/mm^3^	Communication Mode	Bx, By, Bz Magnetic Field Measurement Range	Bits of Data Resolution	Length/mm	Width/mm	Sensitivity of Measurement mt/bit
TLV493D-A1B6	0.1 × 0.73 × 0.65	Serial communication	−130 mt~+130 mt	12-bit	2.9	2.5	0.098

**Table 3:** Color characteristic values of each defect.

	*μ_x_*	*μ_y_*	*μ_z_*	*σ_y_*	*σ_z_*	*S* *_x_*	*S* *_y_*	*S* *_z_*
Defect1	0.18697	0.14017	0.16069	0.01560	0.02427	0.00952	0.00386	0.00820
Defect2	0.18291	0.13998	0.16729	0.01460	0.02359	0.00764	0.00329	0.00740
Defect3	0.17904	0.13925	0.16580	0.01341	0.01973	0.00569	0.00267	0.00497
Defect4	0.17668	0.14004	0.16855	0.01319	0.01922	0.00497	0.00251	0.00445

**Table 4:** Relevant parameters of training samples.

	4 mm	8 mm	12 mm		13 mm
	Hemisphere	Round Table	Hemisphere	Threaded Hemisphere	Hemisphere
80%	1	1	1	1	1
60%	1	1	1	1	1
40%	1	1	1	1	1
20%	1	1	2	0	1

**Table 5:** Relevant parameters of the test sample.

	6 mm	8 mm		10 mm	12 mm
	Hemisphere	Hemisphere	Cylinder	Hemisphere	Hemisphere
80%	1	1	2	1	1
60%	1	1	2	1	1
40%	1	1	2	1	1
20%	1	1	1	1	1

**Table 6:** The result of the gam and sig2 parameters.

	X	Y	Z	XYZ
gam	6.5430	110.6280	31.3698	44.9534
sig2	0.0100	0.0100	0.0100	3.8611

**Table 7:** Recognition rate of test samples.

	X	Y	Z	XYZ
Recognition rate	56.52%	34.7826%	43.48%	82.61%

## Data Availability

Not applicable.
